# Soy and Gastrointestinal Health: A Review

**DOI:** 10.3390/nu15081959

**Published:** 2023-04-19

**Authors:** Damien P. Belobrajdic, Genevieve James-Martin, Darren Jones, Cuong D. Tran

**Affiliations:** Human Health, Health and Biosecurity, CSIRO, Adelaide, SA 5000, Australia; genevieve.james-martin@csiro.au (G.J.-M.); darren.jones@csiro.au (D.J.);

**Keywords:** soy, gastrointestinal, colorectal cancer, short chain fatty acids, microbiome, isoflavones

## Abstract

Soybean is the most economically important legume globally, providing a major source of plant protein for millions of people; it offers a high-quality, cost-competitive and versatile base-protein ingredient for plant-based meat alternatives. The health benefits of soybean and its constituents have largely been attributed to the actions of phytoestrogens, which are present at high levels. Additionally, consumption of soy-based foods may also modulate gastrointestinal (GI) health, in particular colorectal cancer risk, via effects on the composition and metabolic activity of the GI microbiome. The aim of this narrative review was to critically evaluate the emerging evidence from clinical trials, observational studies and animal trials relating to the effects of consuming soybeans, soy-based products and the key constituents of soybeans (isoflavones, soy proteins and oligosaccharides) on measures of GI health. Our review suggests that there are consistent favourable changes in measures of GI health for some soy foods, such as fermented rather than unfermented soy milk, and for those individuals with a microbiome that can metabolise equol. However, as consumption of foods containing soy protein isolates and textured soy proteins increases, further clinical evidence is needed to understand whether these foods elicit similar or additional functional effects on GI health.

## 1. Introduction

Soybean (Glycine max) is the only plant protein that contains all the essential amino acids in amounts that can adequately meet human physiological requirements, and thus it represents an important source of plant protein for both animals and humans. While the majority of soybean protein produced globally (~85%) is used in animal feeds, soybean oil, soy meal and soy-based products including soy milk, tofu, soybeans (edamame) and tempeh also form an important part of many diets [1,2]. Production of soy has doubled in the last decade [1], and is expected to increase further, in line with increasing consumer demand for plant-based protein sources, of which products made from soy, soy protein isolates and textured soy proteins make up a significant part, and global recommendations focused on sustainable dietary patterns [3].

Soybeans are a highly desirable ingredient for producing meat alternatives due to several favourable nutritional properties which set them apart from other commonly consumed beans and pulses (summarised in Table 1 [4]). Soybeans contain around twice the amount of protein compared to other commonly consumed beans and legumes, and are the only plant source that contains all nine essential amino acids. The leucine content of soybeans is comparable to levels found in fish and eggs [5]; leucine has been identified as the most potent amino acid responsible for the postprandial stimulation of muscle protein synthesis, which is important for growth and performance in young people and athletes, as well as maintaining muscle mass in the elderly [6]. Soybeans have a similar dietary fibre content to other commonly consumed beans and pulses [7], mainly in the form of oligosaccharides (predominately stachyose), but also contain non-starch polysaccharides that include pectic polysaccharides, xyloglucan and cellulose [8,9], which have been shown to promote intestinal fermentation [8,10]. Soybeans also contain higher amounts of polyunsaturated fatty acids, including both omega-6 and omega-6 essential fatty acids, linoleic acid and alpha-linolenic acid, than other common legumes [4]. Finally, soybeans are a good source of several micronutrients which are often limited in plant-based diets, including calcium iron and zinc. The high calcium content of soybeans, coupled with the fact that this calcium is efficiently absorbed (in spite of the presence of anti-nutrients such as phytate), makes them particularly amenable to being used as a dairy alternative, and soy milks, cheeses and yogurts are readily available in most global markets [11].

Earlier studies investigating the potential health effects of soybeans and soy products have focused predominately on the role of isoflavones. Given the phytoestrogenic properties of these compounds, it was postulated that high consumption of soy protein could potentially interfere with estrogenic signalling and result in negative health impacts. However, these concerns have not been supported by subsequent scientific studies, and a recent review concluded that soy isoflavones do not interfere with the function of the endocrine system [12]. This review also concluded that soy isoflavone intake did not negatively affect thyroid function, breast, endometrial tissue or oestrogen levels in women, or testosterone or oestrogen levels, sperm or semen parameters in men [12]. There is, however, some, albeit limited, evidence of that soy isoflavone intake is associated with a modest reduction in menopausal hot flushes [13,14,15] and decreased breast and prostate cancer risk [16], although it is not clear whether these effects are due to soy isoflavones or the other nutritional components present in soybeans, such as polyunsaturated fats, fibre, vitamins and minerals. An alternative possibility, and of particular relevance to this review, is that these effects of soy consumption may be a consequence of the beneficial impact of soy and its nutritional components on the function of the GI tract and the intestinal microbiome.

The intestinal microbiome is recognised as playing an important role in the regulation of GI health, and is composed of a diverse consortium of bacteria, archaea, fungi, protozoa and viruses that reside in the digestive tract [17]. The shaping of the gut microbiome starts at birth, influenced by a range of genetic, nutritional and environmental factors including mode of delivery and infant nutrition. The interaction between the GI microbes and intestinal epithelial cells is critical to the protective role of the gut microbiome for the maintenance of health [18]. Conversely, microbial dysbiosis, or a shift in the composition of the microbiome which favours pro-inflammatory species, is associated with the development of a broad range of diseases that includes inflammatory bowel disease, asthma, obesity, metabolic syndrome, cardiovascular disease, immune-mediated conditions, and neurodevelopmental conditions such as autism spectrum disorder [18]. A range of nutritional components of soy milk, soybeans and textured soy proteins escape digestion in the upper digestive tract; these nutrients become a substrate for microbes residing in the large intestine [19]. The nutrients of most interest include proteins, isoflavones and the partially fermentable non-starch polysaccharides such as cellulose, hemicellulose and pectin, along with more rapidly fermented oligosaccharides, raffinose and stachyose [20]. The nutrient content and bioavailability of foods made from soybeans and textured soy proteins varies markedly and depends on the formulation and type of processing methods used [21]; subsequently, this influences how much of each nutrient can potentially reach the large bowel. For instance, the oligosaccharide content of textured soy protein (6630 mg/100 g) is markedly higher than other commonly consumed soy foods such as tempeh, soy cheese and milk (80–550 mg/100 g) [22,23,24]. Additionally, not all dietary protein is completely digested in the small intestine, with approximately 10% of ingested protein reaching the colon each day [25], where it becomes a nitrogen and energy source for specific nitrogen-utilising bacteria, supporting their growth and maintenance in the gut [26]. Fermented soy products also contain microbes which could act as a probiotic if these microbes escape digestion in the upper gut, such as tofu, which commonly contains *Bacillus*, *Enterococcus*, *Streptococcus*, *Lactobacilli* and *Bifidobacteria* [27].

While the effects of soy on the gut microbiota have been reviewed previously [17], and provided some indication that specific soy-based foods could alter the abundance of specific microbes, its effects on additionally important parameters including microbial metabolites and GI health were not reported. Thus, the purpose of this review was to evaluate the current evidence for the beneficial and detrimental effects of soy foods in the diet on GI health. A pragmatic review utilizing a broad search strategy was used to summarise the evidence reported in systematic reviews, umbrella reviews, randomised clinical trials and animal feeding studies for soy milk, soy protein, oligosaccharides and isoflavones.

## 2. Search Strategy

A broad literature search was undertaken to identify all publications that included soy consumption and gut health endpoints. In addition to human studies, reviews and meta-analyses, the literature search also included non-human studies that included animal and in vitro models. The search was carried out in PubMed during June 2021, and the terms used, listed in Supplementary Figure S1, identified 1024 hits. These results were then further sorted into reviews and meta-analyses in one group and other publications in another, and then screened for relevancy, with 20 reviews and meta-analyses and 33 other publications deemed relevant for full-text review and data extraction, and the findings from these studies are included in the review. The effects of soy protein, specific soy-based products and major soy constituents on microbial composition, microbial metabolites and specific GI disease, namely colorectal cancer (CRC), are reported.

## 3. Soy Milk

### 3.1. Soy Milk and the Gut Microbiome

There is some epidemiological evidence that soy milk can modulate the intestinal microbiome, but these effects appear to differ between fermented and unfermented products. A cross-sectional study explored the association of milk (dairy and soy) on the microbiome [28]. They found that high intake of milk and soy beverages was associated with a higher abundance of beneficial faecal microbes including *Faecalibacterium*, *Bifidobacterium*, and *Parabacteroides*; however, no analysis by milk type was performed, therefore it is likely that the differences seen primarily reflect dairy milk intake, given dairy milk is more commonly consumed in America than soy milk. In the three RCTs that have investigated the effect of unfermented soy milk on faecal microbial abundance, the results are inconsistent, with only one study reporting an increased abundance of Lactobacillus after a 2 week intervention period [29]; meanwhile, another study in infants showed no change in any microbes after 4 weeks [30], and a study in overweight and obese men actually reported a decrease in faecal *Lactobacillus* and *Bifidobacterium* abundance after 3 months [31]. In contrast, consumption of fermented soy milk was associated with increased abundance of *Bifidobacterium*, *bifidobacteria* and/or *Lactobacillus* compared to unfermented soy milk in the two RCTs that have evaluated it [29,32]. These findings are supported by studies in rodents, in which the abundance of faecal *Bifidobacterium*, *Lactobacillus* and/or *Bacteroides* was increased in animals fed fermented, but not unfermented, soy milk [33,34]. The greater effects of fermented vs. nonfermented soy milk on the gut microbiome suggests that the microbes present in fermented soy milk may have probiotic effects. There is limited evidence to suggest that nutrients from soy milk reach the large bowel to elicit a prebiotic effect. However, there is one study using a cholesterol-induced colonic inflammation Sprague Dawley rat model which showed that when casein in the diet was replaced with soy milk, it reversed diet-induced dysbiosis by increasing the faecal Firmicutes (*Coprococcus*, *Lactobacillus*, *Blautia* genera) to Bacteroidetes (*Barnesiella* genus) ratio [35].

### 3.2. Soy Milk and Inflammation

Only a small number of clinical trials have evaluated the potential anti-inflammatory properties of soy milk. Two studies, both conducted in patients with type-2 diabetes, showed that circulating levels of C-reactive peptide and cytokines were unaffected by soy milk intake when compared to levels at baseline [36] or compared to cow’s milk consumption [37]. However, studies in rat models of inflammatory bowel disease were inconsistent with this finding, suggesting that soy milk can improve a range of measures of intestinal cell damage and measures of inflammation [38,39]. To confirm whether soy milk may provide benefit to people with inflammatory bowel conditions, a study is currently underway that is investigating whether soy milk consumption (250 mL/d for 4 weeks) by people with ulcerative colitis compared to no dietary intervention [40] improves symptoms and lowers inflammation through mechanisms involving the gut microbiota.

## 4. Soy Proteins

There are no human studies to date that have specifically evaluated the effect of soy proteins on the gut microbiome or GI health, and therefore knowledge in this area is currently based on the results of experimental animal studies. To date, 16 trials, primarily in rodents, have evaluated the effects of soy protein on parameters that include composition of the microbiome, products of microbial fermentation and models of intestinal disease. The majority of these studies have included soy protein at levels comparable to those recommended in human diets and used casein as the control group. However, there is considerable variation between studies in parameters including animal species, strain, age, intervention duration and diet composition (e.g., amount and type of fibre), all of which have the potential to influence microbiome composition, and therefore complicate between-study comparisons.

### 4.1. Soy Protein and Gut Microbiome

Three soy protein dietary intervention studies in rodents have reported higher microbial diversity, considered to be a marker of gut health, and/or altered abundance of specific microbial species in rodents consuming diets containing soy protein concentrate or isolates compared to those fed casein [19,41,42,43,44]. An and colleagues showed that a diet containing soy protein resulted in increased abundance of *Enterococus* and decreased abundance of *Lactobacilli* and *Ruminococus* in rats compared to those fed diets containing fish meal or milk casein [41], whereas another study in rats showed that soy protein-fed animals had a similar abundance of *Lactobacillus* compared to animals fed casein and beef meat [45]. *Lactobacillus* is considered important in host metabolic balance [46,47] and higher abundance may reduce the transfer of antigen load from gut bacteria to the host and alleviate inflammation responses involved with metabolic syndrome [48,49]. Furthermore, when soy protein concentrate or isolate was added to a Western style diet and fed to golden Syrian hamsters [42], the largest differences in microbial abundance were found within the *Bacteroidetes* phylum, with lower relative abundances of *Bacteroidaceae* and *Porphyromonadaceae* than the milk protein-fed groups. The impact of these changes is not clear, particularly given that these changes have not been reported in other studies with a similar design. In contrast, a study in pigs showed that when the animals consumed a diet containing soy protein compared to fish meal or cotton seed protein, there was no change in colonic bacterial diversity [50]. Taken together, animal studies suggest that in comparison to casein, soy proteins can increase microbial diversity and the abundance of specific microbes, but changes vary considerably between studies.

### 4.2. Soy Protein and Microbial Fermentation Metabolites

Animal studies have also evaluated the effect of soy proteins on microbial fermentation products which are known to exert physiological functions, including short-chain fatty acids (SCFAs), and lipopolysaccharides. SCFAs play an important role as a trophic factor and in providing energy for colonocytes [51]. They regulate T regulatory (Treg) cell colonies [52] and also exert beneficial physiological effects on several organs, including the liver, adipose tissue and brain. In contrast, LPS, another product of Gram-negative bacteria, is an endotoxin produced in the large intestine that can enter the circulation and active Toll-like receptors to stimulate inflammation, which has been linked with a range of metabolic health conditions including obesity and type-2 diabetes [53]. Soy proteins have been associated with changes in the production of both these metabolites. In mice and rats, consuming a diet containing soy protein isolate or increasing the amount of soy protein in the diet from 15 to 25% promoted higher faecal levels of SCFAs compared to animals consuming a casein-based diet [43,54]. A study by Bai et al. [55] also showed that when rats consumed a fibre-enriched diet containing soy protein isolate, there was an increase in caecal acetic acid concentration compared to rats fed a fibre-enriched diet containing casein. A small number of studies suggest that soy protein may also stimulate the microbial production of LPS. In comparison to casein, rats consuming soy protein had higher levels of LPS-binding protein in the liver and serum [45,56], and up-regulation of CD14, which is involved in activating macrophages to produce inflammatory cytokines. Although these observations deserve further investigation, genistein has been shown to reduce metabolic endotoxemia in high-fat diet-fed mice [57], and soy increases microbes such as Lactobacillus contribute to the reduction in LPS levels [58]. Together, these findings suggest that soy protein isolates can be utilised by the intestinal microbiome to promote SCFA production, something which is not observed with dairy proteins; however, their effects on LPS and other microbial metabolites is unclear.

### 4.3. Soy Protein and Bile ACID Metabolism

Bile acids are critical components of the GI tract that link the gut microbiota to hepatic and intestinal metabolism and therefore influence GI motility, intestinal permeability and carcinogenesis [59]. The gut microbiota regulates bile acid production and signalling via the biotransformation of intestinal bile acids to unconjugated and secondary forms that readily activate bile acid receptors [59]. There is growing evidence that soy protein consumption modulates bile acid metabolism in the small and large intestine. For instance, a recent study by Wantanabe [60] showed that mice fed a high-fat diet containing soy protein isolate had an enlarged caecal bile acid pool with an elevated secondary/primary bile acid ratio compared to mice fed a high-fat diet containing casein, while faecal bile acid excretion remained unaltered. Soy protein isolate also elicited dramatic changes in the gut microbiome, characterized by an expansion of taxa that may be involved in the biotransformation of bile acids, which is a concern given that there are numerous scientific reports describing bile acids, especially secondary bile acids, as strong carcinogens or promoters of colon cancers [61]. In contrast, another study in a rat model of hepatic steatosis (with Otsuka Long-Evans Tokushima Fatty rats) showed that soy protein isolate added to a Western style diet reduced microbes (*Blautia* and *Lachnospiraceae*) associated with production of secondary bile acids [62]. Thus, at present, the evidence Is limited and inconsistent, and further research is needed to better understand the role of soy proteins in bile acid metabolism.

### 4.4. Soy Protein and Animal Models of GI Disease

A majority of carefully controlled feeding studies (5 out of 8) in rats suggest that diets containing soy protein (>20% weight) may adversely affect a range of measures of GI health compared to dairy proteins. In one study, rats fed diets containing soybean protein for 9 days exhibited higher epithelial cell damage, higher colonic cell proliferation rates and higher faecal water cytotoxicity compared with rats fed diets containing casein as the protein source over this same period [63]. Similarly, another rat study showed that high dietary intakes of soy protein isolate (25%, but not 15% weight) increased the genotoxicity of the colonic luminal environment, resulting in increased colonic DNA damage [54]. Furthermore, majority of rat carcinogenesis models also show that soy proteins increase the number of aberrant crypt foci (ACF; pre-cancerous lesions) or intestinal tumours. Soy protein concentrate (low in isoflavones) increased ACF number [64] but soy products high in isoflavones (soy flour or genestein) lowered total ACF compared to a starch control diet. Furthermore, a study by Gee et al. [65] showed that soy protein increased the colonic ACF number by two-fold when soy protein was consumed prior to carcinogen exposure. Alternatively, colonic ACF decreased when soy protein was consumed for 6 weeks [66], and another showed no effect of high isoflavone soy protein on the number of ACF [67]. In a longer term study that compared four different dietary proteins, animals fed red meat or soybean diets developed a greater pooled number of intestinal tumours per treatment group, and a greater pooled area of tumours compared to animals fed dairy proteins [68].

While of potential concern, it is important to note that any adverse effects of soy protein on GI health may be mediated by dietary fibre within the food matrix (e.g., whole soybean or added fibre in a soy containing food product) or other fibre consumed in the diet [69], since dietary fibre significantly influences how protein is metabolised in the intestinal environment. For instance, a study by Toden et al. [54] showed that when resistant starch was added to the soy protein diet, the level of genotoxic damage to colonocytes was reduced significantly. Another study showed that when xylo-oligosaccharides were added to a human colonic in vitro fermentation system, they lowered the genotoxicity of the fermented material when soy protein was present [70]. This decrease in genotoxicity suggests that dietary xylo-oligosaccharides could potentially lower protein-induced proximal colon genotoxicity and tissue damage in humans. Another study in rats also showed that the combination of soy protein and raffinose substantially increased the caecum IgA concentration, which was not seen with the casein and raffinose group. This favourable change in IgA concentration has been regarded as a positive response to prevent invasion of pathogenic bacteria through the large intestine [55].

## 5. Soy Oligosaccharides and Gut Microbial Activity

The only human clinical study investigating the gut health effects of soy oligosaccharides [71] has provided evidence of a potential beneficial effect, reporting an increase in faecal SCFA and butyric acid levels in humans consuming soy oligosaccharide fibre compared with soy polysaccharide fibre, oat fibre or a self-selected (control) diet. This study also found no differences in whole gut transit time or stool wet weight between the fibre free diet, the soy oligosaccharide, soy polysaccharide fibre and oat fibre diets, suggesting that that soy oligosaccharides have limited effects on bowel function.

A potential beneficial effect of soy oligosaccharides on a range of GI health measures, including microbial abundance and microbial metabolites, is supported by the two animal/in vitro studies that have been conducted to date [32,72]. A pig-feeding trial by Zhou et al. showed that soybean oligosaccharides (0.5 g/100 d diet) increased the concentration of SCFA in the intestinal lumen, increased intestinal microbiota diversity, increased abundance of potentially beneficial intestinal bacteria including *Bifidobacterium* sp., *Faecalibacterium prausnitzii*, *Fusobacterium prausnitzii*, and *Roseburia* [72], and increased a marker of cell wall integrity. Further, the soybean oligosaccharide diet also reduced the numbers of bacteria with pathogenic potential, including *Escherichia coli*, *Clostridium*, and *Streptococcus*, concentrations of several protein-derived catabolites (isobutyrate, isovalerate, and ammonia) and intestinal expression of inflammatory genes (tumor necrosis factor α, interleukin 1β, and interleukin 8 mRNA) in this study. Finally, an in vitro study showed that specific strains of gut microbes such as *Bifidobacteria* can utilise soybean oligosaccharides (raffinose and stachyose), whereas putrefactive bacteria such as *Escherichia coli* and *Clostridium perfringens* cannot [32].

## 6. Isoflavones and the Gut Microbiome

Isoflavones are naturally produced by plants to deter bacterial and fungal infection, and thus have natural anti-bacterial properties. However, it is recognised that only some individuals have microbes that can metabolise specific isoflavones. Daidzein, an isoflavone found abundantly in soy, is converted into equol in the intestine via the action of reductase enzymes belonging to incompletely characterized gut microbes. While all animal species analyzed so far produce equol, only between one third and one half of people (depending on the community) are able to produce equol, ostensibly those that harbor equol-producing microbes [73].

Three human clinical trials were identified that examined how the microbiome of postmenopausal women that metabolise equol respond to soy consumption. A short term (5 day) soy milk (86 mg isoflavone/d) and soy germ (114 mg isoflavone/d) intervention in healthy postmenopausal women [74] showed that faecal microbial changes were dependent upon whether the individuals were able to metabolise equol. This study showed that the strong equol-producer phenotype (18% of study participants) correlated negatively with counts of unfavourable *Clostridium coccoides-Eubacterium rectale,* but positively with the abundance of sulphate-reducing bacteria which are associated with gut inflammation [74]. Another study of similar duration (1 week) showed that in comparison to baseline, consumption of soy bars containing 160 mg soy isoflavones and 1 g saponin [75] increased faecal *Bifidobacterium* abundance. Although there were only 4 of 17 study participants that were equol producers, *Bifidobacterium* and *Eubacterium* were significantly greater in equol vs. non- equol producers. In a third study, gelified soy extract containing isoflavones (100 mg/d) was consumed by 39 postmenopausal women for two months [76]. This study showed that isoflavones alone stimulated dominant microorganisms of the *Clostridium coccoides-Eubacterium rectale* cluster, *Lactobacillus-Enterococcus*, *Faecalibacterium prausnitzii*, and *Bifidobacterium genus*. However, stimulation of the *Clostridium coccoides-Eubacterium rectale* cluster depended on the women’s equol excretion and was transient, with the exception of a prolonged bifidogenic effect, which was less prevalent in equol producers.

Although the published studies do suggest that postmenopausal women who can metabolise equol show more pronounced changes in the microbiome following soy isoflavone intake, the effects are inconsistent because of large study heterogeneity, different levels and sources of isoflavones, different study durations, a limited number of clinical trials and studies with small sample size. Thus, further studies are needed to determine how isoflavones alter the microbiome and their metabolites across a diverse population.

## 7. Soy Food Consumption and CRC Risk

CRC is the third most frequent cancer globally [77,78], and CRC risk is closely related to diet [79]. However, while there is strong evidence that consuming wholegrains, dairy and foods containing dietary fibre reduces the risk of colorectal cancer [16], the role of other dietary components is less consistent, although there have been suggestions from the World Cancer Research Fund that inclusion of soy foods may provide some benefit in reducing CRC risk [16].

An umbrella review of CRC prevention [80] identified three meta-analyses of observational studies that had evaluated the association between soy food intake and risk of GI tract cancer. Of the three meta-analyses, two reported a modest inverse relationship between soy or soybean consumption and CRC risk (RR = 0.85; 95% CI 0.73 to 0.99) [81] (OR = 0.92; 95% CI 0.87 to 0.97) [82], whereas another reported no association [83]. Subsequently, it was concluded that the overall certainty of evidence for soy food reducing CRC risk was very low, with a modest decrease in colon cancer risk [80].

The inconsistency of the protective association of soy or soybean consumption against CRC may be due to various factors potentially causing large study variability/heterogeneity, making it difficult to conclude whether soy products modulate GI cancer risk. The specific limitations include (i) the absence of exact and comprehensive measurement of the consumption of soy foods and/or phytoestrogens; (ii) a limited variety of soy foods consumed, e.g., miso or tofu, which do not thoroughly reflect how phytoestrogens and/or soy are consumed; (iii) the data being obtained from questionnaire-based retrospective studies, which can be highly variable due to deficiencies in the subjects’ ability to accurately recall what they consume [84]; and (iv) potential misclassification, which can also arise from the difference in composition of soy in Asian and Western diets, and can complicate interpretation of the findings. Furthermore, examining the differences between countries, the inverse correlation between soy intake and GI cancer was stronger in countries such as China and Japan compared with the USA. This may relate to higher levels of soy intake and a greater variety in the types of soy consumed [82].

In addition, three meta-analyses were identified that examined the association between key nutrient components of soy, including isoflavones or phytoestrogens, and CRC risk [82,85,86]. Yu et al. [86] assessed the association between soy isoflavone consumption and CRC risk in a meta-analysis that included 13 case–control and four prospective cohort studies. They reported that soy isoflavone consumption was associated with a reduced risk of CRC risk across all groups (relative risk, RR: 0.78, 95% CI: 0.72–0.85), and a sub-group analysis revealed that these relationships were strongest for soy foods/products (RR: 0.79; 95% CI: 0.69–0.89) and in Asian populations (RR: 0.79; 95% CI: 0.72–0.87). The authors concluded that soy isoflavone consumption was most strongly associated with a reduced risk of CRC risk within Asian populations [86]. Consistent with this, the aforementioned meta-analysis published by Tse and Eslick [82] also conducted subgroup analysis for isoflavone intake and showed that compared to total soy consumption (OR = 0.92; 95% CI 0.87–0.97), the inverse association with CRC risk was more pronounced for isoflavone intake (OR = 0.76; 95% CI 0.59–0.98). Another meta-analysis [85] reported an inverse association between phytoestrogen intake and CRC risk; however, this was only significant in case–control studies (pooled n = 6); relative risk (RR) was 0·76 (95% CI 0.69, 0.84) for phytoestrogens and 0·77 (95% CI 0.69, 0.85) for isoflavones (n = 5 studies). This same study also conducted sub-group analysis and reported a dose–response effect for isoflavones, such that for every 20 mg/d increase in isoflavone intake in Asian populations, there was an 8% lower risk of colorectal neoplasms (pooled RR 0·92; 95% CI 0.86, 0.97). As there was large study heterogeneity, Jiang and colleagues [85] concluded that further research was needed.

Another systematic review published over 10 years ago by Jin et al. [87] evaluated the effect of total dietary flavonoid intake on the incidence of colorectal adenoma and CRC. Eight studies (five studies used a prospective cohort design, two were case–control studies, and one was an RCT) with a total of 390,769 participants were included. Overall, the findings were inconsistent, which may have related to the difficulties in measuring flavonoids, given their abundance in a broad range of commonly consumed foods, as well as the wide variation in flavonoids consumed within ethnic subgroups; a high proportion of observational studies included (and most studies were conducted in) Asian populations [87].

### Cell Models Investigating Soy and CRC

Intestinal cancer cell lines have been used to explore the mechanisms underlying the anti-cancer effects of soy protein and its key nutritional constituents. These studies have demonstrated the anti-carcinogenic properties of a wide range of these components, including isoflavones, along with other phytochemicals such as phytosterols, phytates, saponins, protease inhibitors and phenolic acids [88,89], and have suggested several mechanisms of action, including suppression of cell growth [90] and effects on signal transduction such as protein kinase C (PKC) activity [91]. Furthermore, in vitro studies suggest that soy or isoflavones, in particular the primary isoflavone, genistein, may be used in the treatment of existing tumours, either alone or in combination with chemotherapy [92], given that genistein has been shown to induce apoptosis and differentiation in cancer cells and inhibit cell proliferation and angiogenesis [93]. Farina and colleagues (2006) also showed that genistein exhibited antitumor and antiangiogenic activity in B16 melanoma and F3II mammary carcinoma mouse cell lines [94]. The effect of soy isoflavones on CRC cells was reported in one study which showed that Caco-2 cells (a colorectal adenocarcinoma cell line) played a role in the absorption and metabolism of isoflavones’ conjugation [95], but the antitumor or antiangiogenic effects of soy isoflavones on CRC have not been established.

In summary, current epidemiological evidence suggests that there is either a lack of an association or a small inverse association between intake of soybeans or soy foods and CRC risk. However, high intakes of soy isoflavones and phytoestrogens have been associated with a small but significant reduction in CRC risk of between 21 to 24%, particularly in Asian populations. Studies in animal carcinogenesis models tend to suggest that soy proteins may increase CRC risk compared to control-fed animals, but these effects are nullified when fermentable fibers are added to the diet. Although there is some in vitro evidence suggesting that soy isoflavones may have anticarcinogenic properties, there is currently no evidence supporting this effect in colon cancer-derived cell lines. Subsequently, more clinical and mechanistic studies are required to determine whether soy protein and soy isoflavones influence CRC risk.

## 8. Research Gaps

The body of evidence reporting the GI health effects of soy food consumption is limited, and there is a need for large, well-designed randomised clinical trials that address the high variability in microbiome composition between individuals and populations. Additionally, a majority of existing clinical trials have focused on soy milk, which is relatively low in protein content, whereas clinical trials that investigate how textured soy protein foods effect GI health are absent and much needed, given the growing consumer demand for these products. To address the small increased risk in CRC reported in animal models when high levels of soy proteins are consumed in a low-fibre diet, clinical trials that evaluate microbial metabolites and biomarkers of carcinogenesis in intestinal biopsy samples are needed.

There is some evidence suggesting that soy oligosaccharide and isoflavones may have favourable effects on promoting beneficial bacteria and stimulating SCFA production, but a personalised nutrition approach is required to understand whether people that are equol producers gain greater functional benefit, and whether this translates to potential improvements in health beyond the gut.

## 9. Conclusions

In this review, we evaluated and synthesized current evidence relating to the effects of soy-based foods and bioactive components on GI health. The association between soy and/or isoflavone intake and CRC risk has been reported in more than 40 epidemiological studies, and the data suggest there is either a lack of an association or a small inverse association. Of the soy foods consumed, the effect of soy milk on the gut microbiome has received most attention, and current evidence indicates that fermented soy milk, rather than regular soy milk, elicits more consistent changes in the faecal microbiome, most likely due to a probiotic effect rather than the protein or oligosaccharide content of soy milk. Furthermore, the most consistent changes in the faecal microbiome are reported following soy product consumption when individuals are able to metabolise equol. The GI effects of soy protein have been evaluated in animal feeding trials, and although favourable increases in caecal and faecal short chain fatty acids were reported, soy proteins at high levels of intake (>25% weight) induced greater cytotoxic and genotoxic damage to intestinal tissue compared to dairy protein intake. However, some of these effects were shown to be mitigated when fermentable fibres were added to the diets containing soy protein isolates.

In summary, current clinical evidence suggests that some soy foods provide favourable changes in measures of GI health, but further studies are needed to understand whether consumption of foods containing soy protein isolates and textured soy proteins can have similar or additional functional effects on GI health, especially for those individuals with a microbiome that can metabolise equol.

## Figures and Tables

**Table 1:** Nutrient composition of boiled soybeans and selected legumes, per 100 g, cooked [4].

Food Name	Energy, with Dietary Fibre (kJ)	Protein (g)	Total Fat (g)	Total Saturated Fat (g)	Total MUFA (g)	Total PUFA (g)	Linoleic Acid (g)	Alpha-Linolenic Acid (g)	CHO (g)	Dietary Fibre (g)	Calcium (Ca) (mg)	Iron (Fe) (mg)	Zinc (Zn) (mg)
Bean, soya, cooked	614	13.5	7.7	1.12	1.19	4.78	4.19	0.59	1.4	7.2	76	2.2	1.6
Bean, cannellini, cooked	404	6.2	0.6	0.19	0.03	0.26	0.14	0.12	12.2	6.4	46	1.6	0.6
Bean, lima, cooked	355	6.4	0.3	0.07	0.03	0.14	0.1	0.04	10.2	5.3	16	1.3	0.7
Bean, red kidney, cooked	382	7.9	0.5	0.07	0.04	0.28	0.11	0.17	9.1	7.2	34	1.7	1
Chickpea, cooked	466	6.3	2.1	0.21	0.49	0.97	0.93	0.04	13.3	4.7	45	1.8	1
Lentil, green or brown, cooked	595	10	0.8	0.12	0.14	0.4	0.33	0.07	20.2	5.2	21	2.37	0.98
Lentil, red, cooked	458	7.7	0.6	0.09	0.1	0.3	0.24	0.06	16.4	2.2	6	1.44	0.71
Pea, split, cooked	364	6.6	0.4	0.05	0.08	0.19	0.16	0.03	9.1	8.3	13	1	0.6

CHO; carbohydrate, MUFA; monounsaturated fatty acids, PUFA; polyunsaturated fatty acids.

## Data Availability

Not applicable, as new data were created.

## Supplementary Materials

The following supporting information can be downloaded at: https://www.mdpi.com/article/10.3390/nu15081959/s1, Figure S1: Terms list used for literature search.

## References

[1] Ritchie H., Roser M. Forests and Deforestation. https://ourworldindata.org/forests-and-deforestation.

[2] Voora V., Larrea C., Bermudez S. (2020). Global Market Report: Soybeans.

[3] United Nations Department of Economic and Social Affairs The 17 Goals. https://sdgs.un.org/goals.

[4] Food Standards Australia New Zealand (2014). AUSNUT 2011–13—Australian Food Composition Database.

[5] van Vliet S., Burd N.A., van Loon L.J. (2015). The Skeletal Muscle Anabolic Response to Plant- versus Animal-Based Protein Consumption. J. Nutr..

[6] van Loon L.J. (2012). Leucine as a pharmaconutrient in health and disease. Curr. Opin. Clin. Nutr. Metab. Care.

[7] Medic J., Atkinson C., Hurburgh C.R. (2014). Current Knowledge in Soybean Composition. J. Am. Oil Chem. Soc..

[8] Huisman M.M.H., Schols H.A., Voragen A.G.J. (1999). Enzymatic degradation of cell wall polysaccharides from soybean meal. Carbohyd. Polym..

[9] Grieshop C.M., Kadzere C.T., Clapper G.M., Flickinger E.A., Bauer L.L., Frazier R.L., Fahey G.C. (2003). Chemical and nutritional characteristics of United States soybeans and soybean meals. J. Agric. Food Chem..

[10] Tian L.M., Scholte J., Scheurink A.J.W., van den Berg M., Bruggeman G., Bruininx E., de Vos P., Schols H.A., Gruppen H. (2019). Effect of oat and soybean rich in distinct non-starch polysaccharides on fermentation, appetite regulation and fat accumulation in rat. Int. J. Biol. Macromol..

[11] O’Keefe S., Bianchi L., Sharman J. (2015). Soybean Nutrition. SM J. Nutr. Metab..

[12] Messina M., Mejia S.B., Cassidy A., Duncan A., Kurzer M., Nagato C., Ronis M., Rowland I., Sievenpiper J., Barnes S. (2021). Neither soyfoods nor isoflavones warrant classification as endocrine disruptors: A technical review of the observational and clinical data. Crit. Rev. Food Sci. Nutr..

[13] Bolaños-Díaz R., Zavala-Gonzales J.C., Mezones-Holguín E., Francia-Romero J. (2011). Soy extracts versus hormone therapy for reduction of menopausal hot flushes: Indirect comparison. Menopause.

[14] Franco O.H., Chowdhury R., Troup J., Voortman T., Kunutsor S., Kavousi M., Oliver-Williams C., Muka T. (2016). Use of Plant-Based Therapies and Menopausal Symptoms: A Systematic Review and Meta-analysis. JAMA.

[15] Taku K., Melby M.K., Kronenberg F., Kurzer M.S., Messina M. (2012). Extracted or synthesized soybean isoflavones reduce menopausal hot flash frequency and severity: Systematic review and meta-analysis of randomized controlled trials. Menopause.

[16] World Cancer Research Fund, American Institute for Cancer Research (2018). Diet, Nutrition, Physical Activity and Cancer: A Global Perspective. Continuous Update Project Report.

[17] Huang H., Krishnan H.B., Pham Q., Yu L.L., Wang T.T. (2016). Soy and gut microbiota: Interaction and implication for human health. J. Agric. Food Chem..

[18] Brestoff J.R., Artis D. (2013). Commensal bacteria at the interface of host metabolism and the immune system. Nat. Immunol..

[19] Valdes A.M., Walter J., Segal E., Spector T.D. (2018). Role of the gut microbiota in nutrition and health. BMJ.

[20] Gentile C.L., Weir T.L. (2018). The gut microbiota at the intersection of diet and human health. Science.

[21] Rizzo G., Baroni L. (2018). Soy, Soy Foods and Their Role in Vegetarian Diets. Nutrients.

[22] Biesiekierski J.R., Rosella O., Rose R., Liels K., Barrett J.S., Shepherd S.J., Gibson P.R., Muir J.G. (2011). Quantification of fructans, galacto-oligosacharides and other short-chain carbohydrates in processed grains and cereals. J. Hum. Nutr. Diet..

[23] Tuck C., Ly E., Bogatyrev A., Costetsou I., Gibson P., Barrett J., Muir J. (2018). Fermentable short chain carbohydrate (FODMAP) content of common plant-based foods and processed foods suitable for vegetarian- and vegan-based eating patterns. J. Hum. Nutr. Diet..

[24] U.S. Department of Agriculture (2019). Food Data Central.

[25] Cummings J. (1997). Carbohydrate and Protein Digestion: The Substrates Available for Fermentation. The Large Intestine in Nutrition and Disease.

[26] Cummings J., Macfarlane G. (1991). The control and consequences of bacterial fermentation in the human colon. J. Appl. Bacteriol..

[27] Arroyo G., Penas E., Pedrazuela A., Prestamo G. (2005). Intestinal microbiota in rats fed with tofu (soy curd) treated under high pressure. Eur. Food Res. Technol..

[28] Liu Y., Ajami N.J., El-Serag H.B., Hair C., Graham D.Y., White D.L., Chen L., Wang Z., Plew S., Kramer J. (2019). Dietary quality and the colonic mucosa-associated gut microbiome in humans. Am. J. Clin. Nutr..

[29] Cheng I.C., Shang H.F., Lin T.F., Wang T.H., Lin H.S., Lin S.H. (2005). Effect of fermented soy milk on the intestinal bacterial ecosystem. World J. Gastroenterol..

[30] Piacentini G., Peroni D., Bessi E., Morelli L. (2010). Molecular characterization of intestinal microbiota in infants fed with soymilk. J. Pediatr. Gastroenterol. Nutr..

[31] Fernandez-Raudales D., Hoeflinger J.L., Bringe N.A., Cox S.B., Dowd S.E., Miller M.J., Gonzalez de Mejia E. (2012). Consumption of different soymilk formulations differentially affects the gut microbiomes of overweight and obese men. Gut Microbes.

[32] Inoguchi S., Ohashi Y., Narai-Kanayama A., Aso K., Nakagaki T., Fujisawa T. (2012). Effects of non-fermented and fermented soybean milk intake on faecal microbiota and faecal metabolites in humans. Int. J. Food Sci. Nutr..

[33] Wang Z.L., Bao Y., Zhang Y., Zhang J.C., Yao G.Q., Wang S.Q., Zhang H.P. (2013). Effect of Soymilk Fermented with *Lactobacillus plantarum* P-8 on Lipid Metabolism and Fecal Microbiota in Experimental Hyperlipidemic Rats. Food Biophys..

[34] Bedani R., Pauly-Silveira N.D., Roselino M.N., de Valdez G.F., Rossi E.A. (2010). Effect of fermented soy product on the fecal microbiota of rats fed on a beef-based animal diet. J. Sci. Food Agric..

[35] Lee S.-M., Han H.W., Yim S.Y. (2015). Beneficial effects of soy milk and fiber on high cholesterol diet-induced alteration of gut microbiota and inflammatory gene expression in rats. Food Funct..

[36] Feizollahzadeh S., Ghiasvand R., Rezaei A., Khanahmad H., Sadeghi A., Hariri M. (2017). Effect of Probiotic Soy Milk on Serum Levels of Adiponectin, Inflammatory Mediators, Lipid Profile, and Fasting Blood Glucose among Patients with Type II Diabetes Mellitus. Probiotics Antimicrob. Proteins.

[37] Miraghajani M.S., Esmaillzadeh A., Najafabadi M.M., Mirlohi M., Azadbakht L. (2012). Soy Milk Consumption, Inflammation, Coagulation, and Oxidative Stress Among Type 2 Diabetic Patients with Nephropathy. Diabetes Care.

[38] Bitzer Z.T., Wopperer A.L., Chrisfield B.J., Tao L., Cooper T.K., Vanamala J., Elias R.J., Hayes J.E., Lambert J.D. (2017). Soy protein concentrate mitigates markers of colonic inflammation and loss of gut barrier function in vitro and in vivo. J. Nutr. Biochem..

[39] Kawahara M., Nemoto M., Nakata T., Kondo S., Takahashi H., Kimura B., Kuda T. (2015). Anti-inflammatory properties of fermented soy milk with *Lactococcus lactis* subsp. lactis S-SU2 in murine macrophage RAW264. 7 cells and DSS-induced IBD model mice. Int. Immunopharmacol..

[40] Sadeghi O., Milajerdi A., Siadat S.D., Keshavarz S.A., Sima A.R., Vahedi H., Adibi P., Esmaillzadeh A. (2020). Effects of soy milk consumption on gut microbiota, inflammatory markers, and disease severity in patients with ulcerative colitis: A study protocol for a randomized clinical trial. Trials.

[41] An C., Kuda T., Yazaki T., Takahashi H., Kimura B. (2014). Caecal fermentation, putrefaction and microbiotas in rats fed milk casein, soy protein or fish meal. Appl. Microbiol. Biotechnol..

[42] Butteiger D.N., Hibberd A.A., McGraw N.J., Napawan N., Hall-Porter J.M., Krul E.S. (2016). Soy Protein Compared with Milk Protein in a Western Diet Increases Gut Microbial Diversity and Reduces Serum Lipids in Golden Syrian Hamsters. J. Nutr..

[43] Tamura K., Sasaki H., Shiga K., Miyakawa H., Shibata S. (2019). The Timing Effects of Soy Protein Intake on Mice Gut Microbiota. Nutrients.

[44] Le Chatelier E., Nielsen T., Qin J., Prifti E., Hildebrand F., Falony G., Almeida M., Arumugam M., Batto J.M., Kennedy S. (2013). Richness of human gut microbiome correlates with metabolic markers. Nature.

[45] Zhu Y., Shi X., Lin X., Ye K., Xu X., Li C., Zhou G. (2017). Beef, Chicken, and Soy Proteins in Diets Induce Different Gut Microbiota and Metabolites in Rats. Front. Microbiol..

[46] Arora T., Anastasovska J., Gibson G., Tuohy K., Sharma R.K., Bell J., Frost G. (2012). Effect of *Lactobacillus acidophilus* NCDC 13 supplementation on the progression of obesity in diet-induced obese mice. Br. J. Nutr..

[47] Zhang C., Zhang M., Wang S., Han R., Cao Y., Hua W., Mao Y., Zhang X., Pang X., Wei C. (2010). Interactions between gut microbiota, host genetics and diet relevant to development of metabolic syndromes in mice. ISME J..

[48] Zhao L. (2013). The gut microbiota and obesity: From correlation to causality. Nat. Rev. Microbiol..

[49] Cani P.D., Amar J., Iglesias M.A., Poggi M., Knauf C., Bastelica D., Neyrinck A.M., Fava F., Tuohy K.M., Chabo C. (2007). Metabolic endotoxemia initiates obesity and insulin resistance. Diabetes.

[50] Li R., Chang L., Hou G., Song Z., Fan Z., He X., Hou D.X. (2019). Colonic Microbiota and Metabolites Response to Different Dietary Protein Sources in a Piglet Model. Front. Nutr..

[51] Pascale A., Marchesi N., Marelli C., Coppola A., Luzi L., Govoni S., Giustina A., Gazzaruso C. (2018). Microbiota and metabolic diseases. Endocrine.

[52] Smith P.M., Howitt M.R., Panikov N., Michaud M., Gallini C.A., Bohlooly Y.M., Glickman J.N., Garrett W.S. (2013). The microbial metabolites, short-chain fatty acids, regulate colonic Treg cell homeostasis. Science.

[53] Mohammad S., Thiemermann C. (2021). Role of Metabolic Endotoxemia in Systemic Inflammation and Potential Interventions. Front. Immunol..

[54] Toden S., Bird A.R., Topping D.L., Conlon M.A. (2007). Differential effects of dietary whey, casein and soya on colonic DNA damage and large bowel SCFA in rats fed diets low and high in resistant starch. Br. J. Nutr..

[55] Bai G., Ni K., Tsuruta T., Nishino N. (2016). Dietary Casein and Soy Protein Isolate Modulate the Effects of Raffinose and Fructooligosaccharides on the Composition and Fermentation of Gut Microbiota in Rats. J. Food Sci..

[56] Zhu Y., Lin X., Zhao F., Shi X., Li H., Li Y., Zhu W., Xu X., Li C., Zhou G. (2015). Meat, dairy and plant proteins alter bacterial composition of rat gut bacteria. Sci. Rep..

[57] López P., Sánchez M., Perez-Cruz C., Velázquez-Villegas L.A., Syeda T., Aguilar-López M., Rocha-Viggiano A.K., del Carmen Silva-Lucero M., Torre-Villalvazo I., Noriega L.G. (2018). Long-Term Genistein Consumption Modifies Gut Microbiota, Improving Glucose Metabolism, Metabolic Endotoxemia, and Cognitive Function in Mice Fed a High-Fat Diet. Mol. Nutr. Food Res..

[58] Fuke N., Nagata N., Suganuma H., Ota T. (2019). Regulation of gut microbiota and metabolic endotoxemia with dietary factors. Nutrients.

[59] Jia W., Xie G., Jia W. (2018). Bile acid-microbiota crosstalk in gastrointestinal inflammation and carcinogenesis. Nat. Rev. Gastroenterol. Hepatol..

[60] Watanabe K., Igarashi M., Li X., Nakatani A., Miyamoto J., Inaba Y., Sutou A., Saito T., Sato T., Tachibana N. (2018). Dietary soybean protein ameliorates high-fat diet-induced obesity by modifying the gut microbiota-dependent biotransformation of bile acids. PLoS ONE.

[61] Nguyen T.T., Ung T.T., Kim N.H., Jung Y.D. (2018). Role of bile acids in colon carcinogenesis. World J. Clin. Cases.

[62] Panasevich M.R., Schuster C.M., Phillips K.E., Meers G.M., Chintapalli S.V., Wankhade U.D., Shankar K., Butteiger D.N., Krul E.S., Thyfault J.P. (2017). Soy compared with milk protein in a Western diet changes fecal microbiota and decreases hepatic steatosis in obese OLETF rats. J. Nutr. Biochem..

[63] Govers M.J., Lapre J.A., De Vries H.T., Van der Meer R. (1993). Dietary soybean protein compared with casein damages colonic epithelium and stimulates colonic epithelial proliferation in rats. J. Nutr..

[64] Thiagarajan D.G., Bennink M.R., Bourquin L.D., Kavas F.A. (1998). Prevention of precancerous colonic lesions in rats by soy flakes, soy flour, genistein, and calcium. Am. J. Clin. Nutr..

[65] Gee J., Noteborn H., Polley A., Johnson I. (2000). Increased induction of aberrant crypt foci by 1, 2-dimethylhydrazine in rats fed diets containing purified genistein or genistein-rich soya protein. Carcinogenesis.

[66] Helms J., Gallaher D. (1995). The effect of dietary soy protein isolate and genistein on the development of preneoplastic lesions (aberrant crypts) in rats. J. Nutr..

[67] Davies M.J., Bowey E.A., Adlercreutz H., Rowland I.R., Rumsby P.C. (1999). Effects of soy or rye supplementation of high-fat diets on colon tumour development in azoxymethane-treated rats. Carcinogenesis.

[68] McIntosh G.H., Regester G.O., Le Leu R.K., Royle P.J., Smithers G.W. (1995). Dairy proteins protect against dimethylhydrazine-induced intestinal cancers in rats. J. Nutr..

[69] Veettil S.K., Wong T.Y., Loo Y.S., Playdon M.C., Lai N.M., Giovannucci E.L., Chaiyakunapruk N. (2021). Role of Diet in Colorectal Cancer Incidence: Umbrella Review of Meta-analyses of Prospective Observational Studies. JAMA Netw. Open.

[70] Christophersen C.T., Petersen A., Licht T.R., Conlon M.A. (2013). Xylo-oligosaccharides and inulin affect genotoxicity and bacterial populations differently in a human colonic simulator challenged with soy protein. Nutrients.

[71] Kapadia S.A., Raimundo A.H., Grimble G.K., Aimer P., Silk D.B. (1995). Influence of three different fiber-supplemented enteral diets on bowel function and short-chain fatty acid production. JPEN J. Parenter. Enter. Nutr..

[72] Zhou X.L., Kong X.F., Lian G.Q., Blachier F., Geng M.M., Yin Y.L. (2014). Dietary supplementation with soybean oligosaccharides increases short-chain fatty acids but decreases protein-derived catabolites in the intestinal luminal content of weaned Huanjiang mini-piglets. Nutr. Res..

[73] Mayo B., Vázquez L., Flórez A.B. (2019). Equol: A Bacterial Metabolite from The Daidzein Isoflavone and Its Presumed Beneficial Health Effects. Nutrients.

[74] Bolca S., Possemiers S., Herregat A., Huybrechts I., Heyerick A., De Vriese S., Verbruggen M., Depypere H., De Keukeleire D., Bracke M. (2007). Microbial and dietary factors are associated with the equol producer phenotype in healthy postmenopausal women. J. Nutr..

[75] Nakatsu C.H., Armstrong A., Clavijo A.P., Martin B.R., Barnes S., Weaver C.M. (2014). Fecal bacterial community changes associated with isoflavone metabolites in postmenopausal women after soy bar consumption. PLoS ONE.

[76] Clavel T., Fallani M., Lepage P., Levenez F., Mathey J., Rochet V., Serezat M., Sutren M., Henderson G., Bennetau-Pelissero C. (2005). Isoflavones and functional foods alter the dominant intestinal microbiota in postmenopausal women. J. Nutr..

[77] World Health Organization (2002). Cancer Incidence in Five Continents.

[78] Binefa G., Rodriguez-Moranta F., Teule A., Medina-Hayas M. (2014). Colorectal cancer: From prevention to personalized medicine. World J. Gastroenterol..

[79] Deng Y., Wei B., Zhai Z., Zheng Y., Yao J., Wang S., Xiang D., Hu J., Ye X., Yang S. (2021). Dietary risk related colorectal cancer burden: Estimates from 1990 to 2019. Front. Nutr..

[80] Chapelle N., Martel M., Toes-Zoutendijk E., Barkun A.N., Bardou M. (2020). Recent advances in clinical practice: Colorectal cancer chemoprevention in the average-risk population. Gut.

[81] Zhu B., Sun Y., Qi L., Zhong R., Miao X. (2015). Dietary legume consumption reduces risk of colorectal cancer: Evidence from a meta-analysis of cohort studies. Sci. Rep..

[82] Tse G., Eslick G.D. (2016). Soy and isoflavone consumption and risk of gastrointestinal cancer: A systematic review and meta-analysis. Eur. J. Nutr..

[83] Woo H.D., Park S., Oh K., Kim H.J., Shin H.R., Moon H.K., Kim J. (2014). Diet and cancer risk in the Korean population: A meta-analysis. Asian Pac. J. Cancer Prev..

[84] Lechner D., Kállay E., Cross H.S. (2005). Phytoestrogens and colorectal cancer prevention. Vitam. Horm..

[85] Jiang R., Botma A., Rudolph A., Hüsing A., Chang-Claude J. (2016). Phyto-oestrogens and colorectal cancer risk: A systematic review and dose-response meta-analysis of observational studies. Br. J. Nutr..

[86] Yu Y., Jing X., Li H., Zhao X., Wang D. (2016). Soy isoflavone consumption and colorectal cancer risk: A systematic review and meta-analysis. Sci. Rep..

[87] Jin H., Leng Q., Li C. (2012). Dietary flavonoid for preventing colorectal neoplasms. Cochrane Database Syst. Rev..

[88] Fotsis T., Pepper M., Adlercreutz H., Hase T., Montesano R., Schweigerer L. (1995). Genistein, a dietary ingested isoflavonoid, inhibits cell proliferation and in vitro angiogenesis. J. Nutr..

[89] Messina M., Barnes S. (1991). The role of soy products in reducing risk of cancer. JNCI J. Natl. Cancer Inst..

[90] Kim H.-Y., Yu R., Kim J.-S., Kim Y.-K., Sung M.-K. (2004). Antiproliferative crude soy saponin extract modulates the expression of IκBα, protein kinase C, and cyclooxygenase-2 in human colon cancer cells. Cancer Lett..

[91] Tsai C.-Y., Chen Y.-H., Chien Y.-W., Huang W.-H., Lin S.-H. (2010). Effect of soy saponin on the growth of human colon cancer cells. World J. Gastroenterol. WJG.

[92] Fotsis T., Pepper M., Adlercreutz H., Fleischmann G., Hase T., Montesano R., Schweigerer L. (1993). Genistein, a dietary-derived inhibitor of in vitro angiogenesis. Proc. Natl. Acad. Sci. USA.

[93] Polkowski K., Mazurek A.P. (2000). Biological properties of genistein. A review of in vitro and in vivo data. Acta Pol. Pharm..

[94] Farina H.G., Pomies M., Alonso D.F., Gomez D.E. (2006). Antitumor and antiangiogenic activity of soy isoflavone genistein in mouse models of melanoma and breast cancer. Oncol. Rep..

[95] Chen J., Lin H., Hu M. (2005). Absorption and metabolism of genistein and its five isoflavone analogs in the human intestinal Caco-2 model. Cancer Chemother. Pharmacol..

