# Supplementary material for: Soy and Gastrointestinal Health: A Review
Source: Nutrients. 2023 Apr 19;15(8):1959. doi: 10.3390/nu15081959 (PMC10144768; doi:10.3390/nu15081959)
Supplement: Supplementary file 1 [file nutrients-15-01959-s001.zip › nutrients-2329187-supplementary.pdf]

((soy foods[mh] OR soybean proteins[mh] OR soybean oil[mh] OR soy milk[mh] OR isoflavones[mh]) OR (isoflavones[tiab] OR soy[tiab] OR soy-based[tiab] OR soy-rich[tiab] OR soybean[tiab] OR tempeh[tiab] OR miso[tiab] OR natto[tiab] OR edamame[tiab] OR tamari[tiab] OR tofu[tiab] OR bean curd[tiab] OR atsuage[tiab] OR aburaage[tiab] OR TVP[tiab] OR textured vegetable protein[tiab] OR soyfood[tiab])) AND ((Gastrointestinal microbiome[mh] OR Firmicutes[mh] OR bacteroides[mh] OR Fatty Acids, Volatile[mh] OR Phenols[mh] OR Ammonia[mh] OR Gastrointestinal Transit[mh]) OR (Microbiome abundance[tiab] OR Microbiome diversity[tiab] OR Gut microbiome[tiab] OR Gut health[tiab] OR Firmicutes:bacteroides[tiab] OR Bacteria[tiab] OR Microbes[tiab] OR Fermentation[tiab] OR Short chain fatty acids[tiab] OR SCFA[tiab] OR Acetate[tiab] OR Propionate[tiab] OR Butyrate[tiab] OR Para-cresol[tiab] Phenol[tiab] OR Ammonia[tiab] OR Fecal output[tiab] OR pH[tiab] OR transit rate[tiab])) AND (randomized controlled trial[pt] OR controlled clinical trial[pt] OR randomized[tiab] OR placebo[tiab] OR clinical trials as topic[mesh:noexp] OR randomly[tiab] OR trial[ti] OR Meta-Analysis[pt] OR Review[pt] OR Systematic Review[pt] OR Meta-analysis[tiab] OR meta analy*[tw] OR metanaly*[tw] OR metaanaly*[tw] OR narrative review*[tiab] OR systematic review*[tiab] OR umbrella review*[tiab])

Figure S1. Terms list used for literature search.
